# Acute Exacerbations of Chronic Rhinosinusitis

**DOI:** 10.1007/s11882-025-01239-0

**Published:** 2025-12-20

**Authors:** Robert M. Frederick, Kent Lam, Joseph K. Han

**Affiliations:** Department of Otolaryngology – Head & Neck Surgery, EVMS Otolaryngology, Macon and Joan Brock Virginia Health Sciences at Old Dominion University, 600 Gresham Dr. Suite 1100, Norfolk, VA 23507 USA

**Keywords:** Acute, Exacerbation, Chronic rhinosinusitis, Corticosteroids, Antibiotics

## Abstract

**Purpose of Review:**

We aim to highlight recent advancements on the evolving chronic rhinosinusitis (CRS) phenotype: acute exacerbations of chronic rhinosinusitis (AECRS). We focused on studies that expanded the current understanding of its pathophysiology, patient characteristics, and disease burden.

**Recent Findings:**

Defining AECRS has been a topic of discussion for many years. A recent regulatory definition of AECRS in the literature incorporates a > 3 day requirement of worsened symptoms and an escalation of treatment. It is important not to rely on patient-reported rescue medication frequency as it was recently demonstrated these are only obtained for 1/3 of reported AECRS episodes. The pathophysiology behind AECRS is still being evaluated but it appears irritants such as viral insult to the sinonasal microbiome can create a dysbiosis and worsens host immune system breakdown, facilitating a subsequent bacterial infection.

**Summary:**

Many studies are using loose definitions of AECRS because no formal definition has existed until recently. Clinical trials and other studies are relying on patient-reported illnesses, CRS-related antibiotics, and CRS-related corticosteroids to determine an episode of AECRS. Formally defining AECRS is vital in order to conduct future literature on its etiology and clinical outcomes so results may be translatable. Additionally, our review demonstrates that CRS patients with asthma and/or concomitant allergic rhinitis appear to be at an increased risk for developing AECRS and future research should continue to investigate their interplay. Many patients are being overprescribed antibiotics and corticosteroids for reported AECRS episodes. This increases total healthcare spending and increases the risk for adverse effects from corticosteroids and antibiotic resistance. Future research should investigate methods to mitigate this practice.

## Introduction

**Fig. 1:**
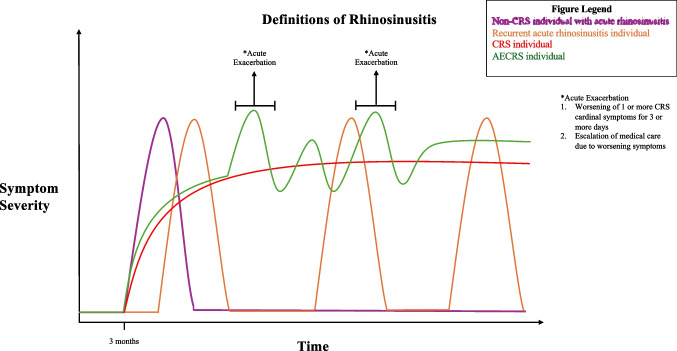
The diagram is representative of chronic rhinosinusitis where there is continuous level of inflammation and at time acute exacerbation

Rhinosinusitis, inflammation within the sinonasal cavities, presents with varied levels of symptoms such as nasal congestion/obstruction, facial pain/pressure, anterior/posterior rhinorrhea, and hyposmia/anosmia. It is classified into acute, subacute, chronic rhinosinusitis (CRS) depending on the time frame – with the latter divided with (CRSwNP) or without (CRSsNP) nasal polyp subgroups. Some patients with CRS may experience acute worsening of their symptomatology for a period of several days, often prompting an escalation of treatment in the form of antibiotic and/or short courses of systemic corticosteroids (SCS), and visit to healthcare provider. This episode of temporary worsening of symptoms, or a loss of control lasting at least 3 days, with a negative impact on quality of life or functionality is deemed an acute exacerbation of CRS (AECRS) in the latest European Position Paper on Rhinosinusitis and Nasal Polyps (EPOS)/European Forum for Research and Education in Allergy and Airway diseases (EUFOREA) [1]. This phenotype of CRS is currently the topic of intense investigation because the level of disease burden it has on patients and the healthcare system. Also, since AECRS definition did not exist until recently, and the pathophysiology behind its occurrence is unclear, further investigation is warranted [2]. In this review, we aim to analyze the latest research on AECRS and to provide an updated summary related to the disease’s pathophysiology, presentation, treatment, and importance for future research. See Fig. 1 for a picture summary on AECRS definition in relation to other CRS definitions.

## Defining AECRS

Currently, no formal definition or grading scale exists for AECRS however there has been regulatory acceptance of AECRS in the REOPEN clinical study [2]. The study defined AECRS as (1) acute worsening of 1 or more cardinal symptoms of CRS lasting at least 3 days, AND (2) escalation of care, defined as initiation of antibiotics or SCS or an unscheduled acute care visit or inpatient care for increased symptoms of chronic (CRS) or acute (ARS) rhinosinusitis. Importantly, exacerbations starting less than 7 days after a previous exacerbation were treated as a single exacerbation. Prior to this accepted definition, many studies – including clinical trials and consensus statements - have used variations that typically revolve around patient-reported worsening symptoms from baseline that prompts the prescription of rescue medications such as antibiotics or systemic corticosteroids. The current versions of this definition lack objectivity and fail to clearly define what symptoms are worsening, to what degree, based on which measurement, and for how long. For example, the International Consensus statement on Allergy and Rhinology: Rhinosinusitis (ICAR:RS) recently defined an acute exacerbation of CRS as an acute and transient worsening of preexisting symptoms in patients with CRS [3]. Although this definition provides framework for defining AECRS, it remains somewhat vague and subjective as the pre-existing symptoms that are worsening are not specified, and the degree to which they are worsened is also not quantified.

A 2020 systematic review by Wu et. al found that there is no consensus definition for quantifying acute exacerbations (AEs), and that definitions are currently largely driven by patient-reported symptoms [4, 5]. One definition based on patient-reported symptoms comes from Walker et. al. who conducted multiple interviews with CRS patients and concluded that patients identify AECRS as worsening sinonasal symptoms that impact sleep, energy level, and lower respiratory tract symptoms [6]. Divekar et. al attempted to add objective measures to identify and better define acute exacerbations (AEs). They reported significantly higher Sino-Nasal Outcome Test (SNOT-22) scores, among other biomarker levels, during patient-reported exacerbations [7]. Increased SNOT-22 scores were found during AEs of cystic fibrosis (CF) patients with concurrent CRS in Zemke et. al study as well [8].

Some have tried to incorporate a duration component into the definition to further clarify. In 2023, Walters et al. interviewed 12 CRS patients to determine a minimum duration of symptoms component to add to a working AECRS definition and published: “a flare up of symptoms beyond day-to-day variation, lasting at least 3 days, and to which a distinct negative impact on a patient's QOL or functionality can be attributed” [1, 9].

Many studies have relied on patient-reported escalation of treatment in the form of CRS-related antibiotic and systemic corticosteroid courses to define AECRS. The European position paper (EPOS) on rhinosinusitis and nasal polyps initially defined AECRS in 2012 as “an acute and transient worsening of preexisting symptoms in CRS patients,” but this was later edited in 2020 to incorporate usage of rescue medications: as, “the worsening of symptom intensity with return to baseline CRS symptom intensity, often after intervention with corticosteroids and/or antibiotics” [10, 11].

Walters et al. summarized the main challenges in creating a standardized definition are that relating the definition to the use of rescue medications is not sensitive enough, symptom based criteria is not specific enough, and retrospective patient reports to define an AE is subject to recall bias [12]. In 2025, Houssein et al. illustrated why we cannot rely on only the frequency of rescue medication usage to define AECRS. This is because they found that, of their 237 sample CRS patients, antibiotics and corticosteroids were prescribed an average of 1.6 times over their 6 month study period compared to the patient-reported average of 4.2 episodes of AE. Meaning that indirect measures such as rescue medication frequency does not capture all episodes of AECRS, only 33% of cases in this study [13]. They also proposed a formal definition to account for this new finding that builds upon their previous definition that included a minimum duration: “a flare up of symptoms beyond day-today variation, lasting at least 3 days, and to which a distinct negative impact on a patients’ quality of life or functionality can be attributed.”

## AECRS as a Metric for CRS Control

More clearly defining AECRS is especially important as many clinical trials use the change in frequency and severity of AECRS as a metric for determining a therapeutic intervention’s efficacy. For example, the NAVIGATE I and II trials’ had outcome measurements related to patient-reported change in baseline symptoms and time to first episode of AE—in which the trial defined an AE as worsening symptoms that required an escalation of treatment [14, 15]. The LIBERTY NP SINUS-24 and SINUS-52 clinical trials tracked AECRS rates by recording the frequency of treatment escalation following dupilumab administration, while SYNAPSE did so for mepolizumab and POLYP 1 and 2 did for omalizumab [16–18].

Other studies have also relied on tracking rescue medication frequency as a proxy of AECRS to determine patients’ response to treatment. Patel et al. investigated the efficacy of several type 2 biologics based on the estimated yearly rates of antibiotic and SCS courses that patients required. They found that patients taking omalizumab, mepolizumab, benralizumab, dupilumab, or reslizumab had a decrease in the yearly rate of antibiotic and systemic corticosteroid use, with a larger reduction seen in those with the most frequent episodes of AECRS [19]. Additionally, Desrosiers et al. demonstrated that Dupilumab reduced the number of CRSwNP patients who required a rescue course of SCS by 75% [20]. Lourijsen et al. used the frequency of AECRS as a metric to compare treatment options, specifically showing that patients who underwent endoscopic sinus surgery (ESS) in addition to medical therapy experienced less AEs and a lower cumulative dose of SCS compared to patients undergoing medical therapy alone [21].

These examples illustrate the importance of creating a formal definition of AECRS, as studies will continue to use its frequency, in the form of rescue medications, as a metric for disease control and treatment efficacy despite Houssein et al. demonstrating that frequency of rescue medications does not capture all of the AE episodes [13].

## Phenotype

Many studies are working to identify characteristics that patients with AECRS have in common in order to better establish the disease’s phenotype. Phillips et al. used the frequency of patient-reported sinus infections and CRS-related rescue medications taken to identify exacerbation-prone individuals. They found that comorbid asthmatics with high sinonasal disease burden were most likely to experience AECRS [22]. Kwah et. Al identified frequent AECRS patients as those who experienced 4+ episodes in 12 months in which a CRS-related antibiotic was prescribed. These frequent AECRS patients had a higher prevalence of asthma, allergic rhinitis, blood eosinophil count > 150cells/µliter, autoimmune disease, and other allergic or immunologic disease [23]. Another study identified high BMI, comorbid asthma, hay fever, and a history of ESS as factors that increased the likelihood of AEs [24]. Patients with migraines and tobacco users were associated with higher AECRS frequency by 29% and 41% respectively in an additional study [13].

A common theme for AECRS patients is an association with comorbid asthma. This relationship is also defined in Banoub et al. study that showed poor asthma control could be predicted by indirect measures of AECRS such as 1 or more patient-reported sinus infections, course of CRS-related antibiotics, or CRS-related corticosteroids [25].

## Pathophysiology

While the exact pathophysiology of AECRS is still under discussion, the 2020 EPOS report summarized potential causes and concluded that it is likely a multifactorial process, that bacterial infection has been over-emphasized in the past, that postoperative changes on the microbiome have a role, and that viral infections likely play a larger role than previously thought [11]. The postoperative changes alluded to are the reduction in biofilm density, as it has been shown to accomplish this without significantly altering the microbiome [26, 27]. The sinonasal microbiome is highly complex and dynamic with constant interplay between environmental pollutants, the host immune system, inflammatory cytokines and interleukins, and normal microbial flora [28]. Specifically, there have been reports of increased IL-5, IL-6, eosinophil major basic protein (MBP), VEGF, and GM-CSF in the nasal secretions of CRSwNP patients during an AE compared to control patients without CRS [7]. However, there may be some confounding with comparing AECRS patients during an AE to a non-CRS control group.

There are normal microorganisms present in the sinonasal microbiome, some advocate that alterations in the ratio of normal to pathologic flora, or any other dysbiosis could contribute to AECRS. Okifo et al. conducted a systematic review that included 14 studies and 1252 individual isolates to characterize the sinus microbiome of AECRS patients [29]. They found the predominant bacteria present during AECRS was *Staphylococcus aureus* and *Pseudomonas aeruginosa. Staphylococcus* and *Pseudomonas* spp, along with *Streptococcus* and *Escherichia* spp, were consistently found in AEs among CRSwNP, CRSsNP, and allergic fungal rhinosinusitis (AFRS) in an additional study studying the microbiome of various CRS types during AEs [30]. Brook et al. concluded that anaerobic bacteria—namely *Peptostreptococcus* spp, *Fusobacterium* spp, gram negative bacilli, and *Propionibacterium* acnes – make up the majority microorganisms found during AECRS as 37% of specimens had only anaerobe isolates compared to 27% with only aerobes, and 37% of samples were mixed aerobes/anaerobes [31]. Additionally, 53% of their specimens had β-lactamase producing bacteria.

Others have attempted to establish the role that viruses play in the microbiome of AECRS. An initial viral infection has been theorized to facilitate later bacterial infection by increasing cellular adhesion molecules, and disrupting epithelial tight junctions [32, 33]. The disrupted epithelium may occur via viral-triggered oncostatin M, or IL-25 expression, both of which are known to increase after viral infections [33, 34]. Kumar et al. conducted a systematic review to elucidate the viral role in CRS that included 6 studies specifically discussing their role in AECRS. There remains to be a clear answer as only 3 of the 6 studies were in favor of a viral trigger for AECRS pointing to evidence of rhinovirus present in the nasal specimens of patients with AECRS [35]. Additionally, as indirect evidence that viruses play a role in developing AECRS, Liu et al. found that Google searches related to rhinosinusitis have consistently peaked during winter months for the past 15 years [36]. Other studies have also demonstrated that the winter is the most common season for an episode to occur [24, 37, 38].

In contrast to the viral etiology theory, one study found no difference in the human rhinovirus expression in CRS patients during an AE compared with controls, and Tan et al. concluded that although there is good evidence to support the role of viral infections causing ARS, it is less clear that viral infections trigger acute exacerbations [7, 39]. Narendran et al. conducted a prospective longitudinal study where they called CRS patients every 2 weeks to collect objective symptoms scores via the Wisconsin Upper Respiratory Symptom Survey (WURSS) and SNOT-22 tests. If scores worsened, patients underwent AECRS assessment where nasal brushings and bacterial swabs were collected. They found 35/80 subjects experienced an AE during the study’s year time frame, predominantly during the Fall or Winter, and that 17/35 AE had bacterial isolates only compared to 8/35 having viral isolates only and 7/35 were mixed infections [38].

The concept of the unified airway, functionally linking the sinonasal cavity and upper airway to the tracheobronchial lower airway, has been theorized for many years, and studies have described a phenotype of AECRS that often involves comorbid asthma [22–25, 40]. The dysbiosis and subsequent abnormal growth and inflammation that occur during chronic obstructive pulmonary disease (COPD) and chronic bronchitis exacerbations may help further explain the pathophysiology behind AECRS [41, 42].

It appears that both viruses and bacteria play a role in the development of AECRS. Viral infections may create a persistent hyper-responsiveness and inflammatory state to the sinonasal mucosa and microbiome via increased expression of deleterious cellular adhesion molecules and/or immuno-epithelial defense disruption, which then leads to an increased susceptibility to bacterial infections that ultimately are responsible for patients’ acute worsening of symptoms.

## Disease Burden

There is substantial literature on CRS’s overall disease burden which negatively impacts patients’ quality of life from both a health and financial perspective [43–45]. Those who experience AECRS are no different, in fact, they may have an increased burden as they must manage baseline CRS symptoms in addition to the intermittent worsening of AEs that typically prompt additional clinic, emergency room or urgent care visits with costly nasal endoscopies, and rescue medication expenses [46, 47]. The average number of lost days of productivity due to CRS in a 3 month period was 1.5 for asthmatics and 2.4 for non-asthmatics in one study [48]. Chu et al. performed a cost utility analysis on treating AECRS and determined that observation was the most cost-effective strategy for managing AECRS if low suspicion of bacterial component [47]. Patients often rely on their daily symptoms in addition to the severity and frequency of AEs when determining disease control and are more likely to escalate treatment by requesting specialist referrals, rescue medication, or seeking out surgery. if they deem their disease uncontrolled [6]. This escalation of treatment undoubtedly raises the financial and time burden of managing the disease. Patients and physicians rely on different metrics to determine the level of disease control. While patients focus mainly on nasal symptoms, physicians consider CRS-related antibiotic or corticosteroid use in addition to nasal and extra-nasal symptoms, per SNOT-22, when determining if disease is controlled or not [49]. The SNOT-22 has been shown to be predictive of AEs, a score > 30 has been associated with at least 1 patient-reported sinus infection or CRS-related antibiotic or corticosteroid use in a 3 month period for both CRSwNP and CRSsNP [50].

Overprescription of antibiotics and SCS increases healthcare spending. A national database study in England of 88,317 cases of CRS found that 80% of patients who received an antibiotic for a CRS diagnosis received a subsequent antibiotic prescription, for which they made an argument to better establish antibiotics’ role in managing CRS to avoid overprescriptions [51]. Proper medical management of CRS has been shown to decrease CRS-related antibiotic and corticosteroid prescriptions; however, patients who previously relied on these rescue medications prior to proper medical management were shown to have continued use [52].

## Treatment

Treating AECRS relies on prevention with consistent use of intranasal corticosteroid rinses and washes, nasal saline irrigations, as first line therapy to maintain baseline CRS symptom burden [53, 54]. Antibiotics and corticosteroids play a large role when AEs do occur; however, their use as “rescue medications” is more so the result of expert opinion and anecdotal evidence as antibiotics’ efficacy has not been supported in the literature – per EPOS 2020, and ICAR 2021 [3, 10]. For example, Sabino et al. found that 2 weeks of amoxicillin-clavulanate did not produce any statistically significant difference in patients’ global sinonasal symptoms, quality of life via the SNOT-22, Lund Kennedy score, or microbiological evaluation compared to placebo when both groups received intranasal corticosteroid and saline washes [55]. In contrast, aspirates and nasal swab samples of CRS patients demonstrated a decreased total abundance of bacterial populations following antibiotic therapy for AECRS [56].

Biofilm production may be limiting antibiotics’ efficacy. Szaleniec et al. investigated the patterns of resistance that patients with AECRS developed. They found that 46% of patients had developed antibiotic resistance, 28% of which were amoxicillin resistant. Fluoroquinolones and aminoglycosides had the lowest rates of resistance. Bacteriophage therapy as an adjunct or replacement to antibiotic treatment for AECRS was discussed, with a reported sensitivity to bacteriophage therapy of 81% [57].

There remains controversy on the utility of repeat cultures for repeat exacerbations. It is common practice for the senior authors of this article to obtain new cultures for each AECRS visit. Yaniv et al. recommended repeating cultures for each exacerbation as their study found a bacterial isolate change in 68% (76/112) of patients, requiring a change in the antibiotic therapy for 40% of patients [58]. In contrast, Yan et al. advocated that culture directed antibiotic therapy was unnecessary as they found no quality of life benefit between culture directed antibiotics and empiric antibiotics [59].

Despite the limited literature on SCS’ efficacy in treating AECRS and no strong recommendation to use them in formal guidelines, they continue to be prescribed. Because they are ubiquitous when discussing the disease, one must stay cognizant of the significant adverse effects that are possible [3]. Waljee et al. described the negative effects associated with short-term (< 30 days, < 20 mg/day per dose) SCS use in their patient population and found an increased rate of sepsis, venous thromboembolism, and fractures that all later diminished in the days to months following the corticosteroid use [60].

## Conclusion

There have been significant strides in determining what microbiota contribute to AECRS as well as delineating it as its own CRS phenotype. Additionally, we support the AECRS definition of worsening of ≥ 1 of the cardinal CRS symptoms for ≥ 3 days prompting patients to seek escalation in treatment; however, there remains no strong recommendation to prescribe systemic corticosteroids or antibiotics for these exacerbations. Future research should concentrate on establishing a more detailed explanation of the disease pathophysiology and creating concrete guidelines for antibiotic and corticosteroid use to limit overprescription of both.

## Key References

- Fokkens WJ, De Corso E, Backer V, et al. EPOS2020/EUFOREA expert opinion on defining disease states and therapeutic goals in CRSwNP. *Rhinology*. 2024;62(3):287–298. 10.4193/Rhin23.415
  - ○ The EPOS2020/EUFOREA paper is a critical citation because it provides the medical community with an expert produced list of definitions for various aspects of care for the CRSwNP patient. Among these, a formal definition of acute exacerbations of CRS is described and is one of the definitions discussed during this review article.
- Houssein FA, Sedaghat AR, Phillips KM. Establishing validity of a novel patient-centered and directly measurable definition of acute exacerbation of chronic rhinosinusitis. *Rhinology*. 2025 Oct 1;63(5):584–590. 10.4193/Rhin24.378
  - ○ This study is important because it demonstrates the fact that patients only receive rescue antibiotics or corticosteroids for 1/3 of their reported exacerbations – highlighting the need for a more precise definition, for which they offered one that includes specific duration parameters and doesn’t only hinge on requiring rescue medications.
- Chu MM, Garcia JT, Sedaghat AR, Scangas GA, Phillips KM. A cost utility analysis for the management of acute exacerbations of chronic rhinosinusitis. *Int Forum Allergy Rhinol*. 2025;15(2):109–119. 10.1002/alr.23452
  - ○ Chu et al. discussed when it was most cost-effective to prescribe rescue medications for AECRS and found that observation was the most cost-effective initial management, unless probability of bacterial infection exceeded 49%. This is important because many clinicians turn to antibiotics as first-line when patients complain of a perceived AE and even many studies’ definitions of AECRS include an antibiotic prescription. This study raises the question of including a turn to rescue medications to define AECRS considering observation is more cost-effective.
- Kumar N, Brar T, Kita H, et al. Viruses in chronic rhinosinusitis: a systematic review. *Front Allergy*. 2023 Dec 5;4:1237068. 10.3389/falgy.2023.1237068
  - ○ This study is important as it is a quality systematic review that reflects the literature’s current understanding of viruses’ role in AECRS pathophysiology, which is still up for debate. It discussed 6 studies that dealt specifically with viruses and AECRS where 3 were in favor of viruses playing an important role and 3 were not in favor. It highlights the fact that there is much to be learned about AECRS pathophysiology still.

## Data Availability

No datasets were generated or analysed during the current study.
